# Design and implementation of a Pacific intervention to increase uptake of urate-lowering therapy for gout: a study protocol

**DOI:** 10.1186/s12939-021-01601-4

**Published:** 2021-12-23

**Authors:** Malakai Ofanoa, Samuela Malakai Ofanoa, Maryann Heather, Siobhan Tu’akoi, Hinamaha Lutui, Nicola Dalbeth, Corina Grey, Bert van der Werf, Felicity Goodyear-Smith

**Affiliations:** 1 Pacific Health Section, University of Auckland, Auckland, New Zealand; 2 Alliance Health Plus, Mount Wellington, Auckland, New Zealand; 3 Department of Medicine, University of Auckland, Auckland, New Zealand; 4 Auckland District Health Board, Auckland, New Zealand; 5 Epidemiology & Biostatistics, University of Auckland, Auckland, New Zealand; 6 Department of General Practice & Primary Health Care, Faculty of Medical & Health Sciences, University of Auckland, PB 92019, Auckland, 1142 New Zealand

**Keywords:** Pacific people, Gout, Co-design, Fa’afaletui, Urate-lowering therapy, Māori, Implementation science, Inequities, Healthcare disparities, Health inequality

## Abstract

**Background:**

Gout is a painful chronic disease which disrupts work and family life and can lead to chronic joint damage. Pacific people in Aotearoa/New Zealand experience significant inequities, with over three times the gout prevalence of the non-Pacific non-Māori populations. Pacific people receive less regular urate-lowering drugs to prevent gout flare-ups, and have nine times the hospitalisation from gout compared with non-Pacific non-Māori people. Rates for Indigenous Māori lie between Pacific and non-Pacific non-Māori. A long-established Collective comprising community members from the Pacific People’s Health Advisory Group, clinical staff from the Pacific Practice-Based Research Network, and University of Auckland researchers have identified that improving Pacific urate-lowering therapy use as the research question of prime importance for improved health outcomes of Pacific people in South Auckland. Building on the existing knowledge, this study aims to develop, implement and evaluate a novel innovative intervention to improve the uptake of urate-lowering therapy by Pacific patients with gout.

**Methods:**

Three-phase mixed methods co-design study using the Fa’afaletui research framework following the STROBE statement. Phase1 is observational times series of prevalence of patients with gout, proportion with urate blood-level monitoring and use of urate-lowering medication over past 5 years. In Phase 2 the Collective will workshop new interventions to address previous uptake barriers, using culturally-appropriate Talanga communications with results synthesised in line with Kakala principles. The designed intervention will be implemented and process and outcome evaluations conducted. Finally, an implementation framework will be produced to facilitate further roll-out.

**Discussion:**

The study aims to enhance health and reduce inequities for Pacific people, contribute to creation of Pacific health knowledge and translation of research findings into Pacific health gains. Potential longer-term impact is a gout-management pathway for use throughout Aotearoa/New Zealand. Māori have similar issues with high gout prevalence and low urate-lowering therapy use hence the intervention is likely to translate to Māori healthcare. The project will contribute to Pacific research capacity and capability-building as well as general upskilling of community and practice members involved in the co-design processes.

**Trial registration:**

The Australian New Zealand Clinical Trial Registry is in process, request number 38206, 1-09-2021.

**Supplementary Information:**

The online version contains supplementary material available at 10.1186/s12939-021-01601-4.

## Background

Gout is a chronic disease of monosodium urate crystal deposition that presents as recurrent episodes of severe acute inflammatory arthritis (gout flares). Gout flares are extremely painful, causing disruption to work and family life, with untreated hyperuricemia leading to chronic joint damage. Pacific people in Aotearoa/New Zealand experience significant inequities, with over three times the gout prevalence of the non-Pacific non-Māori populations: 47% of Pacific men aged ≥65 years have gout, compared with 17% of non-Pacific non-Māori [1]. Flares can be prevented through long-term urate-lowering therapy (ULT) such as allopurinol, which has considerable health and social benefits [2]. In Aotearoa/New Zealand, Pacific people receive less regular urate-lowering drugs (35% versus 44%), and have nine times as many hospitalisations compared with non-Pacific non-Māori people [3]. Pacific people also have an earlier onset, higher flare frequency, more joint inflammation, greater hospitalisation rates and lower health related quality of life than non-Pacific [4]. For Māori, the Indigenous people of Aotearoa/New Zealand, rates fall between Pacific and non-Pacific non-Māori. Despite recognition of this under-treatment, regular use of ULT such as allopurinol among Pacific people remains low [5, 6]. Barriers to regular ULT use include understanding the need to take daily long-term medication; attendances for blood-testing for serum urate levels and for titrating the drug dose; obtaining regular three-monthly repeat prescriptions, and remembering to take daily medication [7]. There are costs in accessing prescriptions and in getting them dispensed, including the requirement for time off work. Such barriers contribute significantly to the inequitable gout outcomes experienced by Pacific people.

The Pacific People’s Health Advisory Group (PPHAG) comprises community members aged in their 20s to 70s from diverse Pacific ethnicities and backgrounds, ranging from young people to the retired. The group was developed after a general practitioner in South Auckland (area with the largest Pacific community in Aotearoa/New Zealand) and a Samoan teacher received Patient and Clinician Engagement (PaCE) training in North America [8, 9]. PaCE is based on the premise that community engagement in generating research questions is necessary for evidence to be translated into best practice to improve health and well-being (the principle of co-design) [10]. A Pacific Practice-Based Research Network (PPBRN) was then set up through the Alliance Health Plus (AH+) Primary Health Organisation (PHO), with designated research officers (general practitioner, nurse or manager) for each member practice. The University of Auckland researchers provided workshop training for both groups in Pacific methodology, and how to identify and ask relevant and important questions which might inform and change practice to benefit Pacific people. The partnership group comprising members of PPHAG, PPBRN, AH+ and University of Auckland researchers is known as the Collective.

Both groups identified improving Pacific urate-lowering therapy (ULT) use as the research question of prime importance for improved health outcomes of Pacific people in South Auckland. Many members of the PPHAG group had personal or family experience of gout and the suffering it can cause. General-practice-based members of the PPBRN had many patients with gout, and identified the non-use of ULT as being a significant clinical problem. This research proposal, based on participatory action research and co-design [11], involves a partnership of researchers and end-users (community members, patients, clinicians) collectively involved in the design, conduct and dissemination of the findings of research [12]. Research that is “carried out with and by local people rather than on them” is an effective means of reducing health disparities [13]. The collaboration extends beyond this specific project, for a long-term synergistic relationship, continuing to build on what has been learnt [14, 15].

The core principles of primary care are a patient-centred equitable approach, providing services that are available, accessible, and affordable [16]. Services need to be comprehensive (caring for the whole person, not just a specific disease), continuous (maintained over time), and coordinated with other services [17]. We add to this that the central tenet of primary care is effective relationships. Relationship is the core value of Pacific people. Quality care depends on good communication between providers and patients, on acknowledging connections, and on engaging in collective decision-making. An effective intervention to improve Pacific uptake and maintain use of gout preventive medication needs to address all these primary care components if it is to reduce the current inequities.

## Methods/design

### Aim and objectives

Building on the existing knowledge, this study aims to develop, implement and evaluate a novel innovative intervention to improve the uptake of ULT by Pacific patients with gout. The objectives are:

1. To determine gout prevalence and management inAuckland practices comparing Pacific, Māori, and non-Pacific non-Māori gout diagnoses, allopurinol prescribing and serum urate testing over a five-year period, and also comparing with data from the three Auckland metro District Health Boards (DHB), as well as national level data.
2. To use a co-design approach to assess ‘what Pacific people think’ (Collective members including community members, clinical staff, PHO workers and other key stakeholders such as people with gout and their families) about possible initiatives to ULT uptake, leading to design of a novel innovative and feasible intervention for South Auckland Pacific patients with gout, and a plan for its implementation.
3. To evaluate the implementation of the plan in a South Auckland context, including its feasibility and acceptability to relevant end-users (especially patients and health care providers), using an implementation science approach.
4. To prepare an implementation framework to guide future implementation roll-out in other Aotearoa/New Zealand settings

### Study design

This is a three-phase mixed methods study using the Samoan research framework *Fa’afaletui* as a culturally appropriate framework for research with Pacific participants. This approach focuses on the importance of considering different perspectives in research, including ‘people at the top of the mountain’ (for example, a national overview) ‘at the top of the tree’ (a regional perspective), who bring long- and middle-distance lenses to the issue, and the ‘man in a canoe fishing’, who is closest to the ‘school of fish’, and most affected by the problem (community members, patients, primary care clinicians) [18].

The intervention will be informed by a stocktake of current Aotearoa/New Zealand initiatives and a systematic review of interventions which found educational campaigns, nurse or pharmacist-led programmes or multi-disciplinary team approaches undertaken in primary care or rheumatology out-patients [19]. None were community-led initiatives, nor conducted outside of traditional healthcare settings. This protocol follows the Strengthening the Reporting of Observational Studies in Epidemiology (STROBE) Statement (Additional file 1).

#### Phase 1: quantitative assessment of Pacific gout health burden and treatment need

The first phase will be an observational times series using routinely collected data to determine the prevalence of patients with gout, and the proportion of these who have their urate levels monitored with blood tests, understanding the need to take daily long-term medication and hence are prescribed ULT. We also wish to assess the proportion who get their prescribed medication dispensed and who receive regular (three-monthly) repeat prescriptions as an indication of taking their daily medication. The measured urate level in the blood is also an indication of whether the patient had been taking their ULT.

We will use the secondary anonymised dataset from the Health Quality and Safety Commission Atlas of Healthcare Variation – gout national and three Auckland metro DHBs (Waitematā, Auckland, Counties Manukau) [1]. In addition, four Primary Health Organisations (PHOs), ProCare, Tamaki Health, Alliance Health Plus (AH+) and National Hauora Coalition), will provide routinely collected clinical data of their de-identified enrolled patient populations. Collectively, these four PHOs have the majority of Pacific and Māori enrolled patients in the three Auckand DHBs. ProCare has 175 practices representing 52.7% of the Auckland DHBs’ population, of whom 22% are Pacific or Māori [20]. Tamaki Health has an enrolled population of 230,000 across 45 Auckland clinics, with 54,700 Pacific patients from South Auckland. AH+ has a network of 40 general practices with a total of approximately 126,000 enrolled patients of all ages, 32% of whom are Pacific and 14% Māori [21], and the National Hauora Coalition has over 120,000 patients in 26 general practices in Auckland Metro, with 22% Pacific and 33% Māori [22].

The study denominator will be all people enrolled with the four PHOs aged 20 years or older at 1 October 2021. The numerator is the number of people diagnosed with gout as coded by the primary care practitioner in the health record. Data extracted include demographic details, gout diagnosis, and prescription and laboratory records pertaining to gout management (see Supplementary material for specific variables). Deidentified data from the PHOs will be transferred via an encrypted USB memory stick and stored on a password-protected University of Auckland drive.

Data will be analysed in R. We will use descriptive epidemiology to determine the prevalence of patients aged 20 years and over with a diagnosis of gout by ethnicity (Pacific, Māori, non-Pacific non-Māori) and gender over the past 5 years. Measurements include percentage of the adult population diagnosed with gout by ethnicity (Pacific, Māori, non-Pacific non-Māori), proportion who have had serum urate monitoring, and hospitalisation for gout. Sub-group analyses will be conducted by age, gender and New Zealand Deprivation Index quintile (NZDep) and time series analyses to determine trends. For the national and DHB samples dispensing, and for the PHOs prescribing, of ULT will be included.

The study population will be described according to gender, age, ethnicity, NZDep decile and whether they have a primary care-coded diagnosis of gout. Continuous variables will be summarised as means with standard deviations and medians with interquartile ranges, and categorical data as frequencies and percentages. The proportion of participants with gout will be compared by ethnicity, gender and 10-year age groups.

Among participants with gout, the proportion prescribed or dispensed ULT, urate test request and result in 2021, 2020, 2019, 2018 and 2017 will be compared by ethnicity, gender and 10-year age groups. The frequency of ULT prescribed over the duration of the gout diagnosis (within the study period) will enable calculation of regularity of three-monthly prescriptions, giving an indication of regular (daily) ULT use. Among participants with gout who have had a serum test result in the preceding 6 months, the proportion with and without serum urate of < 0.36 mmol/L will be compared by ethnicity, gender and age. Differences in proportions between ethnic groups will be assessed using a generalised linear mixed models with binomial or Poisson distribution.

#### Phase 2: designing the intervention and developing the implementation plan

The stocktake of Aotearoa/New Zealand gout programmes, including those not published in the peer reviewed published literature, will be updated. These include Counties Manukau DHB Own My Gout; Northland DHB Gout Stop; and a pharmacist-led clinic. A comparison between the Own My Gout and Gout Stop programmes has recently been reported [23].

A series of workshops will be conducted with Collective members and other key Pacific stakeholders and community representatives to explore their views on interventions currently available, their perceived barriers to Pacific people taking ULT, and to brainstorm innovative alternatives. All PPHAG members, PPBRN research officers, relevant AH+ staff and other relevant stakeholders such as community members and pharmacists will be invited to participate. Participants in the workshops will receive participant information sheets and sign consent forms. Summaries of the interventions identified by the stocktake and systematic review will be produced to show what has already been tried, and what has been effective in different contexts. This will be presented in concise, user-friendly ways, for example in PowerPoint presentations, posters, storyboards or role-plays. The intervention will be co-designed by workshopping with the Collective using qualitative enquiry and nominal group technique where possible to ensure all voices are heard.

Pacific community members, patients and families will be engaged using appropriate cultural processes and protocols. *Talanga* [24] (interactive talk with a purpose) will be used to ensure two-way dialogue takes place when communicating with Pacific people [25]. The *Luva* approach (presentation to others), as exemplified in the *Kakala* research framework [26] will be used to feed back the synthesised material to the Collective group at a subsequent workshop. The novel intervention will be informed based on Pacific people’s holistic view of health as in the *fonofale* model [27]. This model addresses social, physical, mental and spiritual well-being, grounded by family, and overlaid by the Pacific cultural values of relationship, collectivity and collaboration, to create an innovative approach feasible to implement within South Auckland Pacific communities.

Workshops will take place either in-person or via virtual means such as zoom, depending on availability, preferences, and COVID-19 restrictions. Results may be collected on paper and through photographs of whiteboard workings. Key themes may be identified and analysed in NVivo software using a general inductive approach [28]. Suggested interventions will be discussed with study advisers to assess feasibility. Once an intervention has been drafted, the Collective will refine it into a strategy that can be implemented in South Auckland. Factors to be addressed in the intervention include what components it entails (e.g., health promotion, education, prescribing, dispensing, serum urate monitoring, patient reminders, family/whanau involvement), who leads it (e.g., doctor, nurse, pharmacist, team, community-led), and where it takes place (e.g., health premises, community location) and any possible digital modes of delivery (e.g., app, txt messages).

A framework to map the intervention implementation will be developed [29, 30]. A logic model of change [31] will be created using an intervention mapping framework [30]. The logic model will define the inputs (resources, investment needed to implement intervention); key activities (tasks needed to successfully implement the intervention); outputs (measures to be made to demonstrate that activities have been undertaken), and short-term outcomes (changes expected to result).

#### Phase 3 evaluation of the intervention implementation

Precise details of the evaluation will depend on the nature of the novel intervention and its characteristics. The study participants will be individual patients with gout, according to the 2015 ACR/EULAR gout classification criteria [32]. The intervention will be evaluated over a nine-month period (up to three three-month prescribing cycles for ULT). This phase will use an implementation science approach (systematic study of the activities that facilitate successful uptake of an evidence-based health intervention), in this case a strategy and programme to improve ULT amongst Pacific people with gout in South Auckland. The evaluation design will be underpinned by a theoretical framework [29] and informed by behavioural change theory, whereby a person’s attitudes, personal or subjective norms, and their perceived behaviour controls (not doing what they think is wrong) shape an individual’s behavioural intentions, and hence their actual behaviours [33].

Evaluation will focus on:

1. *Process:* This refers to how components of the strategy are delivered or adapted, and how much they conform to the intended intervention components and principles including acceptability and feasibility of intervention delivery. For this study, measures will assess the feasibility of the implementation of the intervention including mechanisms to promote its use to patients with gout, its acceptability, and any enablers and barriers to its use. Patients and family members will be invited to undertake in-depth interviews on their experience of their gout and its management; whether they used the intervention and the enablers and barriers they identify. The *Fonofale model* [27] will be used, exploring how well the intervention met patients’ physical, mental, spiritual, social, family and cultural needs. Acceptability and feasibility data will be sought from personnel involved in providing the intervention. This may be in the form of survey responses (e.g., Likert scale, free text, or both); individual interviewing by phone or zoom, or through focus groups, depending on circumstances and participant preferences.
2. *Mediators of change:* Whether these components reduce perceived barriers, or enhance perceived enablers. Potential data collected include numbers and frequency of intervention delivery, its duration, costings, events that facilitated or impeded its delivery, and other factors dependent on the nature of the intervention. Adaption to real-world circumstances requires a cyclical rather than a liner approach. Iterative changes to the programme delivery may be made in response to feedback and process data analyses during the evaluation period, to improve systematic uptake of the intervention.
3. *Outcomes:* How well the intervention assists patients to take regular ULT. Individual patient data will assess before-after management of gout to determine preliminary effectiveness of the intervention. Data will include gout diagnosis based on gout code classifications as outlined above; regular prescriptions of gout-specific ULT; serum urate testing results during the evaluation period; and hospitalisations for gout.

Analyses will be guided by the evaluation framework RE-AIM [34, 35]. Translation of knowledge into practice requires engagement of all relevant stakeholders, behavioural change, and a flexibility of approach to adapt to real-world contexts. We will evaluate the influences on patient, healthcare professional, and organisational behaviours in the intervention setting to assess whether it can successfully reverse the evidence-practice gap. While the logic model and plan are presented as step-wise and linear, in reality implementation of a complex intervention requires an iterative co-design process, with audits of various components leading to cyclical changes and then being reassessed in a series of feedback loops, and end-users (patients and providers) engaged throughout the process.

Finally, a framework will be produced based on the Consolidated Framework for Implementation Research model [36, 37] which may serve as a guide to extend the implementation to other settings, tailoring the processes and outputs to different contexts. This Framework provides a menu of constructs arranged across five domains (intervention characteristics, outer setting, inner setting, individual characteristics and process) that can provide a practical guide for systematically assessing potential barriers and facilitators, in preparation for implementing an innovation in a particular setting [36]. This will serve as a guide for adaption and implementation of the intervention in other settings.

## Discussion

This study aims to enhance health and reduce inequities for Pacific people, contribute to the creation of Pacific health knowledge and the translation of the research findings into Pacific health gains. The objective of the project is to design a novel innovative and feasible intervention for Pacific people in South Auckland with gout, to increase their allopurinol use. An intervention tailored and targeted by Pacific people, for Pacific people will help reduce health disparities. In the clinical context, the ‘line of sight’ beneficiaries will be Pacific patients with gout, and the general practitioners, practice nurses and community pharmacists collaborating with them in their gout management. Well-managed gout leads to reduced time off work and fewer hospitalisations, hence considerable socio-economic benefits.

A potential longer-term impact is improved management for Pacific people with gout translating this knowledge to a pathway to be used by primary care practices throughout Aotearoa/New Zealand. Although the study focuses on Pacific people, Māori have similar issues with a high prevalence of gout and their ULT uptake could also be improved. These innovative interventions are therefore likely to translate to Māori health care, and possibly to the management of gout patients of other ethnicities. It may also have international implications for populations that experience similar inequitable burdens. The Indigenous Taiwanese population, who are genetically related to Polynesians (Māori and Pacific people), have similar issues with gout [38] and gout is also on the increase in sub-Saharan African countries [39].

The project will contribute to Pacific capacity and capability research gains in Aotearoa/New Zealand as well as general upskilling of the community, practice and PHO members involved in the co-design process.

Gout management is the first of many studies envisaged by the PPHAG and PPBRN of the AH+ to be tackled using a co-design approach, in collaboration with university-based researchers, to answer their questions aimed at improving health outcomes for Pacific people. A second key research question regarding the inequitable rates of rheumatic fever, rheumatic heart disease and resulting poor health outcomes among Pacific people in Aotearoa/New Zealand has been identified and will follow the same protocol outlined in this paper to co-develop an intervention.

## Supplementary Information

**Additional file 1.** STROBE Statement—Checklist of items that should be included in reports of cohort studies.

**Additional file 2.** Variables to be obtained for individual visit data from the PHO.

## Data Availability

Not applicable.

## Abbreviations

AH+: Alliance Health Plus
DHB: District Health Board
GP: General practitioner
NHC: National Hauora Coalition
NZDep: New Zealand Deprivation Index
PaCE: Patient and Clinician Engagement
PHO: Primary Health Organisation
PIS: Participant Information Sheet
PPBRN: Pacific Practice-Based Research Network
PPHAG: Pacific People’s Health Advisory Group
ULT: Urate lowering therapy
