# Supplementary material for: Design and implementation of a Pacific intervention to increase uptake of urate-lowering therapy for gout: a study protocol
Source: Int J Equity Health. 2021 Dec 23;20:262. doi: 10.1186/s12939-021-01601-4 (PMC8696972; doi:10.1186/s12939-021-01601-4)
Supplement: Supplementary file 2 — Additional file 2. Variables to be obtained for individual visit data from the PHO. [file 12939_2021_1601_MOESM2_ESM.docx]

**Supplementary Material: Variables to be obtained for individual visit data from the PHO**

- Patient identifier - AH+ will de-identify data and apply codes to replace National Health Index
- Sex
- Date of birth
- Ethnicity - prioritised
  - Total Pacific (as a binary variable – any Pacific ethnicity reported Y/N)
  - Total Tokelauan (ethnicity group Level 2; binary)
  - Total Niuean (ethnicity group Level 2; binary)
  - Total Tongan (ethnicity group Level 2; binary)
  - Total Cook Island Māori (ethnicity group Level 2; binary)
  - Total Samoan (ethnicity group Level 2; binary)
  - Total Other Pacific people (ethnicity group Level 2; binary)
  - Total Pacific people not further defined (ethnicity group Level 2; binary)
  - Total Fijian (ethnicity group Level 2; binary) *excluding Indo-Fijian
  - Māori (ethnicity group Level 1; binary)
  - European (ethnicity group Level 1; binary)
  - Asian (ethnicity group Level 1; binary)
  - MELAA (ethnicity group Level 1; binary)
- NZ Deprivation (NZDep) index decile
- A current primary care-coded diagnosis of gout (binary)
- Date of gout diagnosis
- Numbers and dates of prescriptions for urate-lowering therapy (allopurinol) in the period 30 September 2016 to 1 October 2021
- Numbers and dates of serum urate tests requested in the period 30 September 2016 to 1 October 2021
- Numbers and dates of serum urate test done (i.e. have results) in the period 30 September 2016 to 1 October 2021
- Serum urate test results in the period 30 September 2016 to 1 October 2021 (actual value in mmol/L)
- Hospital admission(s) with the primary diagnosis of gout
